# Gut microbiota therapy in gastrointestinal diseases

**DOI:** 10.3389/fcell.2025.1514636

**Published:** 2025-02-26

**Authors:** Hanif Ullah, Safia Arbab, Chengting Chang, Saira Bibi, Nehaz Muhammad, Sajid Ur Rehman, Suleman, Irfan Ullah, Inam Ul Hassan, Yali Tian, Ka Li

**Affiliations:** ^1^ Medicine and Engineering Interdisciplinary Research Laboratory of Nursing & Materials, Nursing Key Laboratory of Sichuan Province, West China Hospital, West China School of Nursing, Sichuan University, Chengdu, Sichuan, China; ^2^ Lanzhou Institute of Husbandry and Pharmaceutical Sciences, Chinese Academy of Agricultural Sciences, Lanzhou, China; ^3^ Department of Zoology Hazara University Manshera, Dhodial, Pakistan; ^4^ Hebei Key Laboratory of Animal Physiology, Biochemistry and Molecular Biology, Hebei Collaborative Innovation Center for Eco-Environment, College of Life Sciences, Hebei Normal University, Shijiazhuang, Hebei, China; ^5^ School of Public Health and Emergency Management, School of Medicine, Southern University of Science and Technology, Shenzhen, China; ^6^ Department of Zoology, Government Post Graduate Collage, Swabi, Pakistan; ^7^ Higher Education Department, Civil Secretariat Peshawar, Peshawar, Pakistan; ^8^ Department of Biotechnology and Genetics Engineering, Hazara University, Manshera, Pakistan; ^9^ Department of Microbiology, Hazara University Manshera, Manshera, Pakistan

**Keywords:** gut microbiota, gastrointestinal diseases, cancer, therapy, dysbiosis

## Abstract

The human gut microbiota, consisting of trillions of microorganisms, plays a crucial role in gastrointestinal (GI) health and disease. Dysbiosis, an imbalance in microbial composition, has been linked to a range of GI disorders, including inflammatory bowel disease (IBD), irritable bowel syndrome (IBS), celiac disease, and colorectal cancer. These conditions are influenced by the interactions between the gut microbiota, the host immune system, and the gut-brain axis. Recent research has highlighted the potential for microbiome-based therapeutic strategies, such as probiotics, prebiotics, fecal microbiota transplantation (FMT), and dietary modifications, to restore microbial balance and alleviate disease symptoms. This review examines the role of gut microbiota in the pathogenesis of common gastrointestinal diseases and explores emerging therapeutic approaches aimed at modulating the microbiome. We discuss the scientific foundations of these interventions, their clinical effectiveness, and the challenges in their implementation. The review underscores the therapeutic potential of microbiome-targeted treatments as a novel approach to managing GI disorders, offering personalized and alternative options to conventional therapies. As research in this field continues to evolve, microbiome-based interventions hold promise for improving the treatment and prevention of gastrointestinal diseases.

## 1 Introduction

The gut microbiome consists of bacteria, archaea, viruses, and eukaryotic microbes that colonize the digestive tract. The gut microbiota, which comprises approximately 100–150 times more genes than the human genome, is found in the human intestines and includes approximately 1000 species and 7000 types of bacteria, gram-positive or gram-negative Firmicutes (including the species *Lactobacillus, Eubacterium* and *Clostridium),* and gram-negative Bacteroidetes form the majority of the bacteria (containing *Bacteroides* and *Prevotella*) (Askarova et al., 2020; Blaser, 2017; Flowers and Ellingrod, 2015; Tarawneh and Penhos, 2022). The following five phyla make up the majority of the gut microbial community: Bacteroidetes, Firmicutes, Actinobacteria, Proteobacteria, and Verrucomicrobia (The Human Microbiome Project Consortium, 2012). Individuals’ diet, age, gender, environment, and genes had an impact on the composition of their gut microbiota (Takagi et al., 2019). Dysbiosis of the human gut microbiome has been associated with various pathologies (Perry et al., 2016). Alterations of microbial profiles and density in animals and humans have been associated with the aggregation of brain proteins, neuroinflammation, immune dysregulation, and neuronal and synaptic dysfunction in Alzheimer’s disease (Cryan et al., 2020; Gubert et al., 2020). Proteobacteria and bacteria are involved in maintaining gut microbiota, carbohydrate metabolism, immune modulation and barrier against pathogens (Fan and Pedersen, 2021). Most microbes in the intestine feed from undigested food substrates that have been channeled from the upper digestive system. When bacterial fermentation is carried out on carbohydrates, it does so by using another source of energy and when this is done, what is produced are metabolites that are not good for the human system. Bacterial fermentation of saccharolytic compounds usually results in the formation of beneficial by-products (Rowland et al., 2018).

Gut microbiota in the GI tract is an important component of the host’s health because it controls the cells of the local and distant organs including the brain. The gut-brain axis (GBA) means bidirectional communication between the gastrointestinal tract and the neurological system. This is possible through brain signals, hormones, the immune system, and the gastrointestinal microbiota. Bidirectional transmission in the GBA plays a major role in modulating brain dysfunction, maintaining symbiosis with the host and modulating innate as well as adaptive immune responses The normal gut microbiota synthesizes microbial metabolites and neurotransmitters that interact with host cells including intestinal epithelial cells (IECs) and immune cells. Diet-induced alterations in gut microbial composition and the products generated by the microbiota affect immune-mediated neurological conditions including developmental disorder, neurodegenerative disorder, and emotional dysregulation (Deidda and Biazzo, 2021). The gastrointestinal tract (GI) is the habitat for more than 98% of the bacteria in our bodies. The term “gut microbiota” refers to the particular microorganisms that are present and reside in the gut (Ma et al., 2019) Omics approaches have furthered knowledge of the gut microbiota as a central mediator of the gut-brain axis (Bhattarai et al., 2021). In animal and human models, Gut microbiota can modulate brain behavior and cognitive development by hormones, immunological factors, and metabolites that are produced by gut microbiota and that changing the composition of the microbiota could be a new approach to treatment of brain diseases (Braniste et al., 2014; Lee et al., 2011).

The human microbiome shows early signs of being able to reprogram malnutrition, increase nutrient absorption, and utilize energy from a range of food stuff. Apart from that, microbes play a role in the metabolism of xenobiotics. Various human gut bacteria alter the chemical forms of drugs, toxins, and many insecticides during xenobiotic metabolism (Nakov and Velikova, 2020). The gut microbiota and host immunity are interconnected, complex, and variable. The gut microbiome has been linked to many intestinal and extra-intestinal diseases. Most extensive research into the relationship between gut microbiota and its role has been done in primary GI disorders including IBDs (Lloyd-Price et al., 2019), irritable bowel syndrome (IBS) (Mars et al., 2020), colorectal cancer (CRC) (Tilg et al., 2018), chronic liver diseases (Trebicka et al., 2021) or pancreatic disorders (Riquelme et al., 2019).

In this review, we described the role of gut microbiota in the development and progression of various gastrointestinal diseases, such as inflammatory bowel disease, irritable bowel syndrome, and colorectal cancer. It aims to examine the therapeutic implications of modulating the gut microbiome through approaches like probiotics, prebiotics, and fecal microbiota transplantation. Additionally, the review seeks to highlight emerging strategies for personalized microbiome-based treatments and their potential to improve the management of GI disorders. Ultimately, it aims to provide insights into the future directions of microbiome research for disease prevention and treatment.

## 2 Gut microbiota dysbiosis and associated disease

Gut dysbiosis has been linked to numerous diseases, including autoimmune disorders, neurodegenerative diseases, metabolic conditions, and inflammatory diseases. This microbial imbalance can impair immune function, affect brain health, and elevate systemic inflammation, all of which contribute to the development of various health issues (Ullah et al., 2023). Studies indicate that disruptions in the composition and quantity of gut microorganisms are associated with immune dysfunction, protein misfolding in the brain, inflammation, and altered neuronal and synaptic development. Growing evidence from modern epidemiological, physiological, and omics research, as well as cell and animal model studies, underscores the critical role of the intestinal microbiota in both health and disease (Cryan et al., 2020; Ullah et al., 2024). The intestinal microbiota has been shown to play a major role in both health and disease based on the findings of current epidemiological, physiological, and omics-based investigations as well as cellular and animal studies (Ding et al., 2019). Even though the research on the composition of the complex gut microbiota is still extremely limited and there is no knowledge about most of the functional features, some promising studies indicate that they suggest an incredible potential for the radical improvement of both the existing treatment methods and the understanding of the nature of diseases (Ding et al., 2019; Punia Bangar et al., 2022; Rajoka et al., 2021). Various researches have given supporting data in support of the hypothesis that the gut microbiota controls immunity, energy balance, obesity, and obesity-related diseases (Piccioni et al., 2022).

**FIGURE 1:**
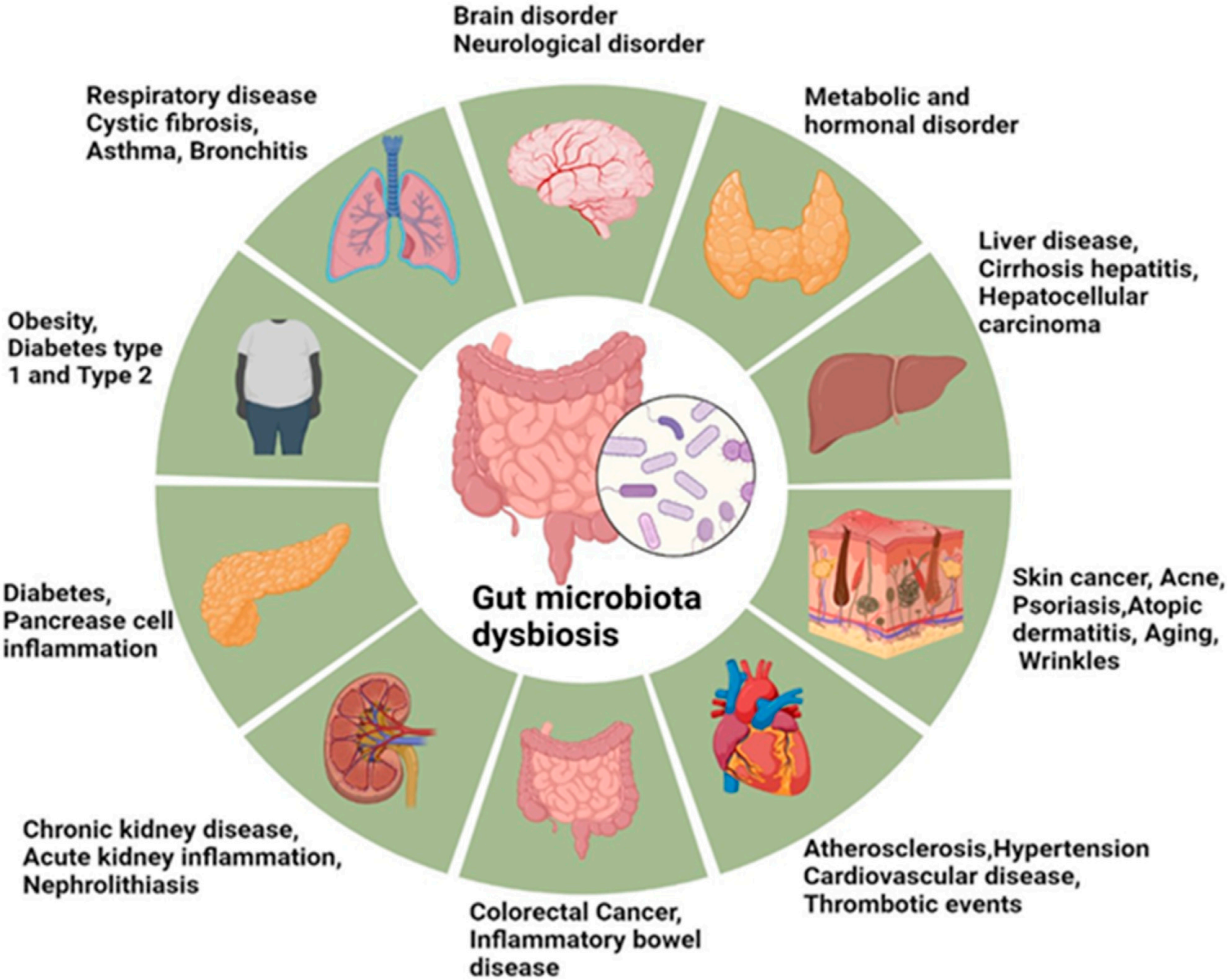
Gut microbiota dysbiosis and associated disease.

Sensitively, food supplements and diets influence the variation of microbial flora in the gut. Low-quality diets, high in fat content, are associated with diabetes, metabolic syndrome, and obesity states that manifest significant alterations in the composition of gut microbiota. Shifting the circadian physiological clock of the body raises the chances of developing intestinal dysbiosis, which in return leads to inflammatory and metabolic diseases including cancer, diabetes and intestinal inflammatory diseases (Reynolds et al., 2017). Similarly, several nonalcoholic fatty liver diseases (NAFLDs), IBDs, hepatocellular carcinoma, cardiovascular diseases (CVDs), alcoholic liver disease (ALD), chronic kidney diseases (CKDs), and cirrhosis are associated with the gut microbiota and its metabolites (Hsu et al., 2020; Philips et al., 2022; Toumi et al., 2021). The gut microbiota modulates the development and function of immune cells within the central nervous system such as microglia; and impacts on physical immune cells which exert influence on the immune response of the central nervous system (Fung et al., 2017). The gut microbiota is a diverse system that plays major roles in the healing of tissues, metabolism of nutrients, and protection against infections (Thursby and Juge, 2017). Enteral nutrition lacks sensory properties in foods but can shape intestinal bacteria to control inflammation. Research findings prove that imitating feeding increases salivation secretion, enhances the gastrointestinal motility, removes oral bacteria and viruses, and also alleviate depressive disorder (Chapman et al., 2019; Roslan et al., 2020). The gut microbiota communicates with and controls, host microbial symbiosis through neural, endocrine humidoral, immunological, and metabolic signaling systems. Nonpathogenic microorganisms of the human gut, belonging to a variety of bacterial genera, have a mutual interaction with their host and help in the immune system and protection against pathogens. Several human diseases and conditions have been linked to dysbiosis of the gut microbiota including anxiety and depression, hypertension and cardiovascular diseases, obesity and type 2 diabetes mellitus, inflammatory bowel diseases and cancer as shown in Figure 1.

## 3 GI tract, gut microbiota, and human metabolism

The gastrointestinal (GI) tract, also known as the digestive system, is an essential organ system responsible for processing food and eliminating waste. It includes the mouth, esophagus, stomach, small intestines, large intestines (colon), and anus. The proper functioning of the GI tract is crucial to overall health, and any disorders can significantly impact an individual’s quality of life. Common conditions that affect the GI tract include IBS, gastroesophageal reflux disease (GERD), IBD, peptic ulcer disease, gastroenteritis (inflammation of the stomach and intestines), and various forms of cancer (Ullah et al., 2024).

The intestine is composed of several layers: the mucosa, submucosa, muscularis propria and serosa. Such vital part of the body as mucosa embraces epithelium, lamina propria, and muscular mucosa in the context of the microbiome (Jaladanki and Wang, 2016). The deepest epithelial layer is bound by tight junctions, adheres junctions and desmosomes from outside to inside (Camilleri, 2019). These layers limit the movement of intestinal contents beyond the gastrointestinal tract but has different states of Patho physiologies. The mucosa and gut epithelial cells form the barrier against endotoxemia and other infections. Probiotics influence organ and intestinal permeability, with short-chain fatty acids (SCFAs) and secondary bile acids gaining attention for their key roles in gut health. These metabolites, produced by gut microbiota, help modulate immune function by regulating gut immunity. SCFAs and metabolites like inosine—derived mainly from *Bifidobacterium* and *Akkermansia muciniphila* stimulate naïve T cells, promoting Th1 differentiation and enhancing their effector activity. This highlights the critical role of microbiota-derived metabolites in immune regulation (Mager et al., 2020). It is hypothesized that changes in gut microbiota can lead to a “leaky gut,” increasing intestinal permeability and allowing harmful pathogens, toxins, and microbial products to enter the bloodstream. This triggers an immune response. Therefore, the regulation of the immune system by gut microbiota is essential for maintaining biological integrity and preventing disruptions in intestinal barrier function (Stevens et al., 2018; Thevaranjan et al., 2017). Many identified metabolites, such as those containing sulfur and phenol, are known to be toxic to enterocytes, disrupt intercellular tight junctions (TJ), and stimulate bacterial translocation, all of which support this idea (Cox et al., 2014). These effects cause inflammatory diseases, perturbation of immune cell function, and the failure to clear highly pathogenic organisms (van der Poll et al., 2017). The intestinal microbiota plays a crucial role in the maturation and development of the immune system during the early life. Disruption of the microbiota-gut-brain axis can lead to various medical, psychological, and neurological disorders (Cryan et al., 2019).

The human microbiome performs a wide range of metabolic functions that are vital for the proper functioning of individual enzymes in the gut lining, liver, or anything else that concerns the host. Next, gut microbiota modulates the host’s health by affecting the fate of the nutrients circulating. Given the well-establish immunological involvement of gut microbiota in humans, efforts have been made to determine the functions of individual microbes in context of the pathways involved in metabolism of nutrients (Cardona and Roman, 2022). The human gut microbiota is associated with the breakdown of ingested Fibres, Proteins and Peptides through fermentation as well as Anaerobic bacteria/Yeast action (Yadav et al., 2018). Proteins and fats are the other major alimentary constituents fermented by gut microbiota, mainly simple sugars and carbohydrates. Fibers specifically, from Bacteroidetes and Firmicutes phyla, ferment in the colon to branched-chain and SCFAs, lactate, ethanol, hydrogen and carbon dioxide; the products are then either metabolized or expelled by the host (Patrascu et al., 2017). Acetate, propionate, and butyrate are the primary short-chain fatty acids (SCFAs) found in human feces, typically in a molar ratio ranging from 3:1:1 to 10:2:1. This ratio is consistent with values observed in the intestine, including in cases of early sudden death. These SCFAs serve various crucial functions in the body, with butyrate being particularly valuable as the main energy source for colonocytes (Rauf et al., 2022). Of these, butyrate is of immense value to humans because it is the colonocyte’s primary energy source (Wang M. et al., 2019). It also have anticarcinogenic effects since it inhibits cancer cells’ proliferation especially colon cancer through apoptosis and controls genes expression by inhibiting the histone deacetylase activity (Havenaar, 2011). Propionate is yet another important energy supply with an important function in the gluconeogenesis of PPCs, especially of the liver epithelial cells (Cani, 2018). Acetate cofacilitates the growth of other bacteria; for example, *Faecalibacterium prausnitzii* cannot be cultivated axenically in a medium without acetate (Rowland et al., 2018). The human gut microbiota also actively produces vitamins such as B7 (biotin), B9 (folate), and K as well as breaking down and inactivating inherent human-generated carcinogens such as pyrolysates (Selber-Hnatiw et al., 2017). The data indicate that bacterial metabolites entering the circulation can substantially alter the host metabolisms via interactions with appropriate host membrane or nuclear receptors (Bhutia et al., 2017).

Intestinal TJ proteins are involved in the regulation of barrier status which defines how the composition of the microbiota in the gut interacts with the immune cells. TJ proteins include occludin, claudin and JAMs which act as barrier to the intercellular space of the epithelial cells of the intestine and regulate the paracellular transport of molecules in ions (Ullah et al., 2024). Changes in TJ proteins, whether through degradation, dephosphorylation, or displacement, lead to a compromised barrier controlling the passage of toxins, undigested food particles and microorganisms through the epithelium and to elicit an immune response. Such increased permeability can also stimulate immune cells such as the macrophages and dendritic cells to release inflammatory cytokines, and enhance antigen presentation for possible autoimmune conditions (Ullah et al., 2024). Since the permeability can be altered, the composition of the gut microbiota can change affecting growth and survival of population. This can lead to cytokine production for inflammation which, if released, augments permeability, producing a cycle of inflammation. On the other hand, normal healthy intestinal epithelial barrier, due to intact TJ proteins, does not allow increased microbial translocation and immune activation. This preserves a diverse composition of gut microbiota, strengthens immunotolerance, and increases anti-inflammatory cytokine secretion to protect against excessive inflammation.

## 4 Gut brain axis

Merging research on the gut-brain axis with gastrointestinal conditions such as IBS and IBD can provide valuable insights into the complex interplay between gut microbiota and the central nervous system (CNS). The gut-brain axis refers to the bidirectional communication between the gut and brain, which is mediated through neural, immune, and endocrine pathways, and is increasingly recognized for its role in gut disorders. Dysbiosis, or microbial imbalance, is believed to disrupt this axis, leading to alterations in gut motility, pain sensitivity, and immune responses, which are common in conditions like IBS and IBD. Studies have shown that changes in gut microbiota composition can influence the brain via the vagus nerve, production of neurotransmitters such as serotonin, and systemic inflammation, contributing to both gastrointestinal symptoms and psychological comorbidities like anxiety and depression (Ullah et al., 2023). For instance, IBS patients often exhibit alterations in gut microbiota, which can affect the central nervous system’s regulation of pain and mood, further exacerbating their symptoms. In IBD, dysbiosis has been linked to an increased production of pro-inflammatory cytokines, which may influence both intestinal and brain inflammation (Shaikh et al., 2023). Understanding the mechanisms by which gut microbial imbalances affect CNS function could not only provide new therapeutic targets for IBS and IBD but also offer a more holistic approach to treating these diseases, integrating both gastrointestinal and neurological aspects of health (Ullah et al., 2023).

## 5 Gut microbiota and gastrointestinal diseases

The gut microbiota has been increasingly linked to the development and progression of various cancers, including colorectal cancer. Dysbiosis, or an imbalance in the microbial composition, can influence carcinogenesis by affecting immune responses, inflammation, and the metabolism of carcinogenic compounds. Specific bacterial species have been shown to produce metabolites that either promote or inhibit tumor growth. Furthermore, alterations in the gut microbiome can impact the effectiveness of cancer therapies, highlighting the potential of microbiome modulation as a therapeutic strategy. Understanding the complex relationship between gut microbiota and cancer may lead to novel approaches for prevention, early detection, and treatment (Kim and Lee, 2022).

The review highlights the current understanding of the immune system, gut microbiota, and their potential roles in cancer immune surveillance. Tumor suppression in humans relies on the immune system, but metastatic tumor cells can evade immune detection by using checkpoints like CTLA-4 and PD-1/PD-L1, thereby inhibiting immune responses and weakening anticancer immunity.This version simplifies and streamlines the information for better readability and clarity (Buchbinder and Desai, 2016; Qin et al., 2019). Moreover, CD8^+^ tumor-infiltrating T cells enhance the expression of PD-1 and CTLA-4 in patients with pancreatic ductal adenocarcinoma (PDAC) (Bailey et al., 2016; Wartenberg et al., 2018). When CTLA-4 is blocked, there is an average 50% decrease in *Bacteroidales* and *Burkholder ales* but an average 2.3-fold increase in Clostridial bacteria. Some of the studies show that inhibition of PD-1 in cancer patients reacts with gut microbiota and some species like *Faecalibacterium, Bifidobacterium, Akkermansi*a, etc (Derosa et al., 2022; Routy et al., 2018). PD-1 inhibition in cancer patients has been linked to the gut microbiota, including *Faecalibacterium, Bifidobacterium,* and *Akkermansia,* according to several studies (Wu et al., 2009), direct DNA damage (Arthur et al., 2012), and the induction of cholesterol synthesis (Tsoi et al., 2017). Alteration in the composition of gut microbiota may result in malignant neoplasms including PDAC and CRC. A large number of researches have been devoted to the investigation of differences in gut microbiota composition between cancer patients and healthy individuals. Regarding microbiota, in individuals with cancers other than prostate, colorectal, or ovarian malignancies, Proteobacteria, the number of the Firmicutes phylum, and Actinobacteria were increased compared to healthy donors (Kostic et al., 2012; Pushalkar et al., 2018; Shen et al., 2010). In contrast, healthy donors had higher Bacteroidetes levels than did patients with PDAC, CRC, ovarian cancer, or breast cancer (Wu et al., 2020). Increased levels of *Ak. muciniphila, E. coli, P. copri, Alistipes putredinis, Ruminococcus torques,* and *Prevotella* are associated with dysbiosis in CRC patients (Ma et al., 2021).

### 5.1 Gut microbiota and gastric cancer

The gut microbiota plays a significant role in the development of gastric cancer by influencing inflammation, immune responses, and the metabolism of carcinogens. Dysbiosis, particularly an overgrowth of certain bacterial species like *Helicobacter pylori*, is strongly associated with increased gastric cancer risk. Microbial imbalances can also impact the tumor microenvironment, promoting tumorigenesis (Wang et al., 2023). Currently cancer as the second most common cause of death globally (Fitzmaurice et al., 2017). Gastric cancer remains one of the most frequent malignancies in the world; the overall prevalence of gastrointestinal malignancies is about one-third of all cancer diagnoses globally (Siegel et al., 2021). It has pathogenesis with environment factors, *H. pylori* infection and genetics. There is compelling evidence pointing directly to *H. pylori* as a major actor in gastric carcinogenesis, although the position of other microbes present in the stomach requires further evaluation (Ilie et al., 2011). *H. pylori* infection leads to a state of chronic infection with activation of molecular remodeling in the target gastric glandular epithelial cells leading to gland atrophy, intestinal metaplasia and GC (Correa, 1992). Research conducted on microbial imbalance linked to gastric adenocarcinoma development remains inconclusive on the change patterns of gastric microbial community. Research done on microbial ecology has shown that microbial richness is substantially decreased in inflammatory diseases and cancer (Gong et al., 2016). Recent research suggests that other microbes, in addition to *H. pylori*, may play a role in the etiology of gastric cancer (GC). Bacteria that are adapted to the hypo acidic environment of the stomach can contribute to carcinogenesis through various mechanisms, such as the production of toxic metabolites, induction of inflammation, alterations in stem cell behavior, and enhanced cell proliferation. These microbial interactions may promote the development and progression of GC (Petra et al., 2017). A comparative analysis of the stomach microbiota of gastric cancer patients was performed in a relatively recent study and it was found that there were similarities between the microbiota of gastric cancer patients, dyspepsia patients and those with normal gastric mucosa (Dicksved et al., 2009). Ferreira et al. demonstrated that gastric carcinoma is associated with a significant reduction in *Helicobacter* abundance, along with an increase in the relative abundance of genera such as *Lactobacillus*, *Citrobacter*, *Achromobacter*, *Clostridium*, and *Rhodococcus*. Interestingly, *Phyllobacterium*, a bacterium typically found in plant roots, was found to be enriched in gastric carcinoma samples, suggesting a potential novel microbial association with gastric cancer (Ferreira et al., 2018). In another study, differences in microbial composition between early and late gastric cancer were not identified, although microbial density was shown to be reduced from normal peritumoral tissue, and tumoral tissue (Liu et al., 2019). Moreover, *Prevotella copri* and *Bacteroides uniformis*, and the number were decreased; in contrast, *Prevotella melaninogenica, Streptococcus anginosus,* and *Propionibacterium acnes* numbers were increased in tumor samples compared to the normal and peritumoral samples (Liu et al., 2019). One research that offered the details on the microbial profile in relation with diverse subtypes of GC was contemplated recently. Fusobacteria, Bacteroidetes, and Patescibacteria were enriched in signet-ring cell carcinoma, whereas Proteobacteria and *Acidobacteria* were enriched in adenocarcinoma (Ravegnini et al., 2020). The study of gut microbiota and gastric cancer is the need for more longitudinal studies to establish causal links between microbiome alterations and cancer development. Additionally, the mechanisms through which specific microbial species influence gastric carcinogenesis remain poorly understood. There is also a lack of comprehensive research on how microbial diversity affects treatment outcomes and the response to therapies in gastric cancer patients. Addressing these gaps could lead to better diagnostic and therapeutic strategies.

### 5.2 Inflammatory bowel disease

Inflammatory bowel disease (IBD), including Crohn’s disease and ulcerative colitis, have been strongly linked to imbalances in gut microbiota. Dysbiosis, characterized by a reduction in beneficial microbes and an overgrowth of pathogenic bacteria, is commonly observed in IBD patients. These microbial alterations can contribute to inflammation, immune dysregulation, and tissue damage in the gut. Crohn’s disease and ulcerative colitis, the two main forms of IBD, are widespread conditions (Khor et al., 2011). Historically, IBD has been most common in developed countries in Europe and North America. However, in recent years, the incidence of IBD has been rising rapidly in newly industrialized regions, including parts of Asia, the Middle East, Africa, and South America. This shift is thought to be related to changes in lifestyle, diet, and environmental factors associated with industrialization (Kaplan and Ng, 2017). IBD is comprised of inflammatory bowel diseases of the appendix and rectum and mostly from Crohn’s disease and ulcerative colitis. The exact cause of IBD, however, still has not been determined; however, in genetically predisposed individuals, it develops after the immune system’s overreaction to normal stimuli, like food or the bacteria and other organisms that are overgrowth in the digestive system (Khor et al., 2011). A meta-analysis by Pittayanon et al. (2020) included 48 studies comparing the gut microbial composition of IBD patients with healthy controls. In Crohn’s disease (CD), the abundance of *Christensenellaceae* (Firmicutes phylum), *Coriobacteriaceae* (Actinobacteria phylum), and *F. prausnitzii* was reduced, while *Actinomyces*, *Veillonella*, and *Escherichia coli* were found to be increased compared to healthy individuals. In ulcerative colitis (UC), *Eubacterium rectale* and *Akkermansia* were decreased, while *E. coli* was elevated. The study concluded that, overall, the gut microbiota composition in IBD patients was either less diverse or similar to that of healthy controls (Pittayanon et al., 2020). In fact, the intestinal microbiota has been recognized to be involved in IBD development for the past few years. It emerges from multiple lines of evidence that gut microbiota plays a critical function in regulating intestinal inflammation. Most studies found that IBD patients have reduced inter-visibility of the gastrointestinal tract microbiomes (Matsuoka and Kanai, 2015). One of the key findings regarding gut microbiota shifts in IBD patients is a dramatic reduction in the abundance of *Firmicutes* and *Proteobacteria*. The decrease in microbial diversity in these patients is primarily attributed to the significant reduction in *Firmicutes*, particularly within the *C. leptum* group, which includes *F. prausnitzii*. These reductions were found to be significantly lower in IBD patients compared to healthy individuals (Wang et al., 2014). It is worth to note that most of the human pathogenic bacteria belonging to the phylum Proteobacteria, which was found to be involved in the complication of IBD more and more (Mukhopadhya et al., 2012). Microbial diversity analysis reports an overall rise in species from this phylum, evidence supporting the idea that it is involved in IBD-induced persistent inflammation (Hold et al., 2014). Additionally, there is an increased mean relative abundance of *Ruminococcus gnavus* in IBD patients, particularly when compared to healthy controls. This shift suggests a potential microbial signature associated with the disease (Ortqvist et al., 2019).

### 5.3 Colorectal cancer (CRC)

Colorectal cancer (CRC) is closely linked to alterations in the gut microbiota, with certain bacteria promoting carcinogenic processes. Dysbiosis, characterized by an imbalance of beneficial and harmful microbes, may contribute to inflammation, DNA damage, and tumorigenesis in the colon. Pathogenic bacteria like *Fusobacterium nucleatum* and *Streptococcus bovis* have been implicated in CRC development (Fusco et al., 2024). CRC is one of the most common cancers and is a major global health burden. CRC ranks third in terms of incidence and second in mortality worldwide, accounting for 1.8 million new cases and 881,000 deaths in 2018 (Sung et al., 2021). Colorectal carcinogenesis is influenced by both host factors and microbial factors. Key contributors include lifestyle choices and dietary habits. For instance, pre-illness dietary factors such as high fat intake, reduced consumption of fiber, excessive red meat, alcohol, and low levels of short-chain fatty acids (SCFAs) have been associated with an increased risk of adenomas and CRC. These dietary patterns may promote an environment that favors carcinogenic processes in the colon (Willett et al., 1990). Some bacteria promote carcinogenesis by releasing substances that can damage DNA. Examples include reactive oxygen species produced by *Enterococcus faecalis*, increased nitric oxide levels from immune cells triggered by *Helicobacter hepaticus*, and enterotoxins released by *Enterotoxigenic Bacteroides fragilis* (ETBF), which activate the *c-MYC* oncogene (Shiryaev et al., 2013). Other species, such as *Parvimonas micra*, *Solobacterium moorei*, and *Peptostreptococcus anaerobius*, have also been strongly associated with CRC. Tsoi et al. specifically reported higher levels of *P. anaerobius* in both tumor lesion biopsies and stool samples from CRC patients, compared to healthy individuals (Tsoi et al., 2017). Yu et al. observed that, unlike bacteria, fungal alpha-diversity did not show significant variation between CRC patients and healthy controls, although the fungal compositions were disrupted. Notably, the ratio of *Basidiomycota* in the PVR was significantly higher in CRC patients compared to healthy individuals. Additionally, the class *Malasseziomycetes* was enriched in CRC patients, while the classes *Saccharomycetes* and *Pneumocystidomycetes* were found to be depleted in the CRC group (Coker et al., 2019). Although there are differences in the composition of the intestinal microbiota between patients with CRC and healthy individuals, several bacterial species have been implicated in CRC development. One such bacterium is *Streptococcus bovis* (*S. bovis*), a gram-positive *coccus,* which has been consistently associated with CRC. Its presence in the gut microbiota may be linked to the promotion of carcinogenic processes in the colon (Boleij et al., 2011; Srivastava et al., 2010). Enterotoxigenic *B. fragilis*, a bacterium producing *B.fragilis* toxin (BFT), causes diarrhea and inflammatory bowel disease (IBD) (Chung et al., 2018). *Fusobacterium nucleatum* is found to be enriched in human colorectal adenomas and carcinomas. Its increased presence in the gut microbiota has been associated with colorectal tumorigenesis, suggesting that *F. nucleatum* may play a role in the development and progression of colorectal cancer by influencing inflammation, immune response, and cellular processes within the colorectal tissue (Kostic et al., 2013), and may contribute to disease progression from adenoma to cancer (Bashir et al., 2015). Another human study conducted in the current year showed that *F. nucleatum* is enriched in individuals with early-stage CRC (Liu et al., 2020). Colorectal cancer (CRC) is primarily associated with a group of bacteria rather than a single pathogenic microorganism, where the harmful effects of these microbes outweigh the beneficial roles of commensal bacteria. Conversely, certain beneficial bacteria are found in lower abundance in CRC patients. These include probiotics such as *Clostridium butyicum*, a butyrate producer, and *Lactobacillus* and *Streptococcus thermophilus*, which produce lactate. These bacteria are believed to have anticancer properties, potentially helping to prevent CRC by fostering a healthier gut microbiota, producing beneficial metabolites, and supporting colorectal health.

### 5.4 Irritable bowel syndrome

Irritable bowel syndrome (IBS) has been linked to alterations in gut microbiota composition. Studies suggest that IBS patients often exhibit dysbiosis, with an imbalance in microbial diversity and shifts in the abundance of certain bacteria. Key changes include a reduction in beneficial microbes like *Firmicutes* and an increase in pathogenic bacteria such as *E. coli*. These microbial alterations are thought to contribute to IBS symptoms like bloating, abdominal pain, and altered bowel movements by affecting gut permeability, immune response, and gut-brain signaling (Cheng et al., 2024). Although the initiation of IBS is complex, newer concepts on the mechanism of IBS point directly to dysbiosis of normal flora being involved in the low-grade inflammation in the gut that characterizes the syndrome (Brint et al., 2011). Most experts believe that gut microbial dysbiosis participates in the development of IBS through enhancing pathogen attachment to the wall of the bowel (Ghoshal et al., 2012). The use of phylogenetic microarrays and qPCR analysis and showed that there was a definite separation between the GI microbiota of the IBS patients and the control group. In IBS, Firmicutes became higher, especially *Ruminococcus, Clostridium,* and *Dorea,* while the amount of *Bifidobacterium* and *Faecalibacterium spp*. (Rajili Stojanovi et al., 2011). Likewise in a cross-sectional study comparing IBS pediatric patient to a healthy control using qPCR for Firmicutes and Proteobacteria, a higher *Dorea, Ruminococcus,* and *Haemophilus parainfluenzae* were observed. Thus demographic factors and bacterial profiles of pediatric IBS patients were significantly different from the healthy ones: *Bacteroides* depleted, while Alistipes enriched; nevertheless an augmentation of the latter was related to more frequent pain (Saulnier et al., 2011).

### 5.5 Non-alcoholic fatty liver disease

Non-alcoholic fatty liver disease (NAFLD) is increasingly linked to gut microbiota imbalances, with dysbiosis playing a crucial role in its development and progression. Alterations in gut microbial composition can influence liver metabolism, inflammation, and fat storage, contributing to NAFLD. Specific microbial taxa, as well as microbial-derived metabolites, have been implicated in modulating liver function and disease severity (Safari and Grard, 2019). NAFLD is a significant chronic condition that, if left untreated, can lead to liver cirrhosis and liver cancer. The integrity of the gut barrier is essential to prevent the translocation of intestinal microflora to the liver. A meta-analysis revealed that oxidative stress (OS) in NAFLD patients was elevated and positively correlated with the severity of hepatic steatosis (De Munck et al., 2020). Also, the present study further showed that there are distinctions in the gut microbiota between NAFLD patients and healthy controls. However the presence of *Lactobacillus*, Dorea, and *Streptococcus* were higher in the gut of NAFLD patients while the concentration of *Rumenococcus, Prevotella,* and *Flavobacterium* was down in their gut (Jiang et al., 2015). Fecal transplantation experiments revealed that when mice were infected with stool from NAFLD patients they developed hypertriglyceridemia in the liver and developed liver steatosis and change of gut microbes are implicated in NAFLD (Hoyles et al., 2018), the end products of saccharolytic and proteolytic fermentation by the gut microbiota (GM) are believed to influence the gut-liver axis and can directly impact the pathogenesis of non-alcoholic fatty liver disease (NAFLD). Saccharolytic fermentation generates short-chain fatty acids (SCFAs) like acetate, propionate, and butyrate, which promote gut health and influence liver metabolism, potentially mitigating fat accumulation. On the other hand, proteolytic fermentation leads to the production of amino acid derivatives and toxic metabolites such as ammonia and hydrogen sulfide, which may exacerbate inflammation and liver injury. These microbial metabolites, by interacting with the gut-liver axis, can either promote or protect against NAFLD depending on the microbial balance and fermentation pathways (Wang et al., 2018). Metabolites produced at these fermentations interfere with the gut–liver axis in line with NAFLD pathogenesis (Canfora et al., 2019). Butyrate improves intestinal barrier function by boosting the expression of uncoupling protein 2 (UCP2), tight junction proteins, and mucus in the epithelial layer. Additionally, it helps alleviate liver injury in ob/ob mice, highlighting its potential role in enhancing gut health and protecting the liver (Kelly et al., 2015). The GM also suppresses hepatic lipid synthesis through BA and modulating the gene expressed through the FXR signaling pathway (Iruzubieta et al., 2020). Further, NAFLD is believed to be associated with glycine usage directly. Glycine effectively ameliorates experimentally induced NAFLD through modulation of fat utilization, blood metabolites, gut microbiome, and protective antioxidant, glutathione (Rom et al., 2020). Additionally, fibroblast growth factor 15/19 (FGF15/19), a hormone stimulated by bile acids during the late fed phase, inhibits hepatic lipogenesis. It does so through an epigenetic mechanism, where FGF15/19 interacts with its target nuclear receptors, such as small heterodimer partner (SHP), which then recruits DNA methyltransferase-3a (DNMT3a) to the lipogenic gene loci, including *FASN*. This epigenetic regulation leads to the suppression of lipogenesis in the liver (Kim et al., 2020). In future research on NAFLD and gut microbiota should focus on identifying specific microbial biomarkers for early diagnosis and disease progression. Understanding the mechanistic pathways linking dysbiosis to liver inflammation and fibrosis is crucial. Additionally, exploring microbiome-based therapies for NAFLD treatment and prevention remains a key research gap. Table 1 demonstrates that imbalances in the gut microbiome can lead to various health issues within the gastrointestinal tract.

**TABLE 1:** Gut microbiota dysbiosis and gastrointestinal disease.

Disease	Bacteria that decreases in number	Bacteria that increases in number	References
Colorectal cancer	*Prevotella, Ruminococcus spp., Pseudobutyrivibrio remains*	*Acidaminobacter* *Phascolarcto bacterium* *Citrobacter farmer*	Khoruts et al. (2010), Wang et al. (2019a)
Colon cancer	*F.prausnitzii*	*Akkermansia muciniphila*	Khoruts et al. (2010), Wang et al. (2019a)
Gastric cancer	*Eubacteriumrectalie*	*Clostridium„Fusobacterium*	Khoruts et al. (2010), Wang et al. (2019a)
IBD:Chron’sdisease	*Bacteroides, Faecalibacterium prausnitzii, Bifidobacterium adolescentis*		Kassam et al. (2013)
Ulcerative cholitis	*Bifidobacteria, Roseburiahominis, Faecalibacterium prausnitzii* *Lachnospiraceae, Ruminococcaceae*		Kassam et al. (2013)
Liver disease	*Alistipes, Bilophila,Veillonella* *Faecalibacterium, Ruminococcus, Bifidobacterium, Prevotella* *Coprococcus, Veillonellaceae* *Prevotellacopri, Faecalibacterium* *Haemophilu*	Claustridum, Bacteroidetes Betaproteobacteria *Lactobacillusspp.,Collinsella, Corynebacterium, Prevotellaceae* *Ruminococcaceae* *Sarcina, Sutterellaceae*	Paramsothy et al. (2017)
IBS	Bifidobacterium Faecalibacterium *Bacteroides*	Firmicutes: Bacteroidetes ratio Ruminococcus *Dorea, Clostridium* *Gammaproteobacteria(pIBS)* *Haemophilus influenzae* (IBS)	Ghoshal et al. (2012)
IBD	bacterial diversity Firmicutes, Bacteroidetes *Lachnospiracheae* *Clostridium leptumand coccoides* group *(Faecalibacterium prausnitzii*) Roseburia Phascolarctobacterium	bacterial numbers in mucosa (CD), Gamma–proteobacteria Enterobacteraceae adherent invasive *Escherichia coli(CD), Clostridium spp.*	Morgan et al. (2012)
Crohn’s Disease	*Faecalibacterium prausnitzii*, *Roseburia*	*Akkermansia muciniphila*, *Bacteroides fragilis*	Vich Vila et al. (2018)
Non-Alcoholic Fatty Liver Disease (NAFLD)	*Bacteroides spp.*, *Lactobacillus*	*Firmicutes* (e.g., *Roseburia*, *Akkermansia muciniphila*)	Vallianou et al. (2021)
Peptic Ulcer Disease	*Lactobacillus*, *Bifidobacterium*	*H. pylori*	Guerra-Valle et al. (2022)

## 6 Therapy of gastrointestinal disorders

The gut microbiota plays a crucial role in human gastrointestinal health and the development of various diseases. As such, targeting the gut microbiota offers a promising approach for managing chronic gastrointestinal conditions. Strategies for manipulating the microbiota include the use of prebiotics, administration of probiotics, fecal microbiota transplantation (FMT) for bacterial recolonization, and antimicrobial treatments to eliminate harmful pathogens or optimize specific bacterial populations. Several pharmacological interventions aimed at addressing dysbiosis in gastrointestinal patients, including probiotics, have been explored. Probiotics may be more effective for individuals whose symptoms remain unresolved with current treatments. The underlying theory suggests that probiotics help alleviate symptoms by promoting the restoration of a healthier gut microbiota or through the beneficial metabolites they produce (Nishida et al., 2022). Probiotics are active and specific microscope organisms that enhance the composition of the host’s microbial flora with desired effects (Schrezenmeir and de Vrese, 2001). *Lactobacillus* and Bifidobacterium species that are everyday popular probiotic bacteria have mucosal trophic effects including stimulation of epithelial barrier function, inhibition of pathogen adhesion, regulation of existing microbiota and modulation immune system components (Shanahan, 2010; Sheil et al., 2007). A previous study on a different strain, Bifidobacterium breve CCFM1025, also demonstrated improvements in both gastrointestinal and psychological symptoms in patients with major depressive disorder (MDD) (Tian et al., 2022). Lactobacilli and Bifidobacteria are also acknowledged probiotic strains regulating gut and mental health (Burnet and Cowen, 2013). Because of psychoactive content of these strains is therefore coined as psych biotics because of its ability to alter behaviors in individuals with depression or anxiety (Burnet and Cowen, 2013; Liu et al., 2016). Tao et al. track down the events through which the probiotic *Lactobacillus* GG releases soluble molecules that in turn activate the p38 MAPK pathway that synthesizes heat-shock proteins to shield the intestinal epithelial cells from damage (Tao et al., 2006). It revealed that VSL#3, a high potency probiotic medical food which contains eight strains could induce ulcerative colitis (UC) Some other probiotics, such as *Bifidobacterium bifidum* as well as *L. acidophilus* have been used in UC in order to manage the condition (Shen et al., 2014; Sood et al., 2009). Some other probiotics, such as *B. bifidum* as well as *L. acidophilus* have been used in UC in order to manage the condition. (Kato et al., 2004), and *L. reuteri* ATCC55730 (Oliva et al., 2012), have also been associated with positive outcomes in IBD patients according to previous reports. Furthermore, the benefits of probiotics have been linked to the restoration of goblet cell quantity and function as well as the induction of protective immunoglobulin secretion by the mucosal immune system in the intestinal tract, including secretory IgA, protective defensins, and bactericidins (Nami et al., 2014). The gut microbiota regulates the metabolism and side effects of irinotecan (CPT-11), a topoisomerase inhibitor prodrug of SN-38 that is frequently used to treat colorectal cancer (Wallace et al., 2018). Pre- and probiotic products which are currently presented as food supplements such as the normally referred to as psych biotics must adhere to the quality regulations of WHO classifications. Such requirements entail confirmation that the formulations used contain well-defined microbial strains for the intended use; probable human clinical trial results; scientifically rationally formulated or conforming to local/national authority specifications; and, most importantly, remains viable and effective at the recommended dose during storage. (Binda et al., 2020; Simon et al., 2021). Unique challenges in multi-omics include fluctuations in kappa values due to differences in software platforms and the reliance on a limited number of globally accessible, high-quality microbiome databases. This dependence restricts the available data to well-characterized microbes, transcripts, proteins, and metabolites, limiting the breadth of insights that can be gained from less studied or newly discovered entities (Chetty and Blekhman, 2024). Highlighting specific probiotic strains and their clinical efficacy would strengthen the review. For instance, *Saccharomyces boulardii* and *Lactobacillus rhamnosus* have shown effectiveness in reducing symptoms of irritable bowel syndrome (IBS) and preventing relapses of *Clostridium difficile* infection. In inflammatory bowel disease (IBD), *E. coli* Nissle 1917 and *Lactobacillus plantarum* have demonstrated promise in maintaining remission and improving gut health (Rau et al., 2024).

Phage therapy is an emerging treatment for gastrointestinal diseases, particularly those caused by bacterial infections, by utilizing bacteriophages viruses that target and kill specific bacteria. This approach offers a targeted alternative to antibiotics, which can disrupt the gut microbiota and contribute to antibiotic resistance. Phage therapy has shown promise in treating conditions like *C. difficile* infection, as well as foodborne bacterial infections such as *Salmonella* and *E. coli*. It works by using phages to specifically target and eliminate pathogenic bacteria while leaving beneficial gut microbes intact, potentially restoring microbial balance and reducing inflammation. However, its clinical use is still in early stages, with ongoing studies focused on optimizing phage selection, delivery methods, and safety (Ranveer et al., 2024).

Fecal microbiota transplantation (FMT) is an emerging therapeutic approach that involves transferring stool from a healthy donor to a patient, aiming to restore a balanced gut microbiota, particularly in gastrointestinal diseases associated with dysbiosis. FMT has demonstrated significant success in treating recurrent *C. difficile* infection (CDI), reducing relapse rates and restoring microbial diversity. It is also being explored for conditions like inflammatory bowel disease (IBD), including Crohn’s disease and ulcerative colitis, as well as irritable bowel syndrome (IBS), though with mixed results. The procedure helps restore gut homeostasis, strengthen the intestinal barrier, and modulate the immune system, potentially influencing metabolic pathways and reducing inflammation (Tian et al., 2024).

**FIGURE 2:**
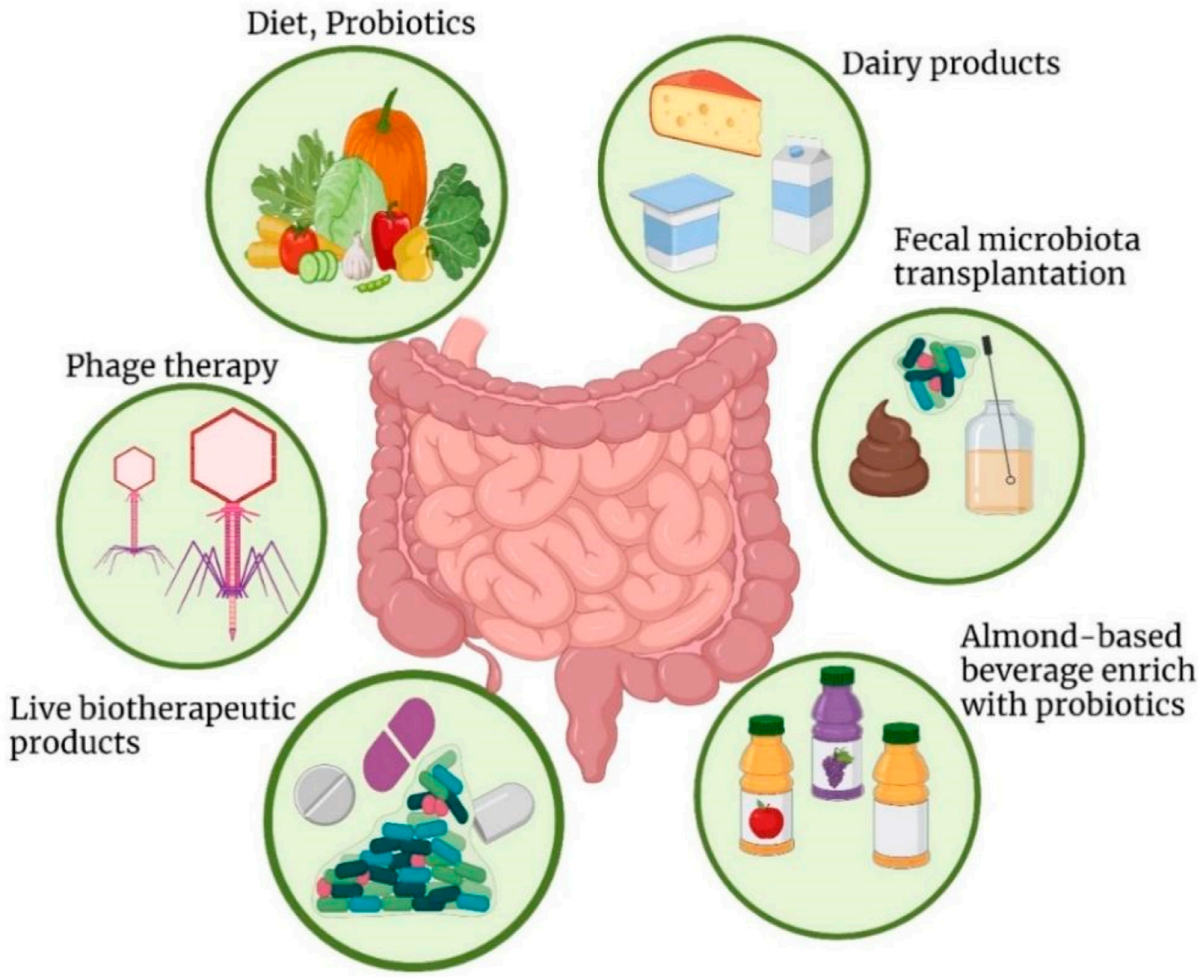
Strategies to modify the gut microbiota for disease treatment include incorporating dairy products fortified with probiotic strains, such as yogurt, milk, and cheese, which are well-known for promoting digestive health and may also support mental wellbeing. Probiotics are available in various forms, including powders and microencapsulated options, to enhance their effectiveness. To increase the availability of probiotics and bioactive compounds, probiotic-enriched fruits, vegetables, and beverages have been developed. These probiotic-infused foods not only help maintain gut homeostasis but also influence brain function.

Gut microbiota research is subjected to the following main challenges mostly affecting elaborative revelation of the relationship between resident bacteria and the host. Several studies have not gone further to dissect the human microbiota in terms of composing the genetic material of the microbes, proteins, and metabolic pathways that maybe essential in understanding the etiopathogenetic position of the microbiota (Filardo et al., 2024). However, there are significant challenges associated with the current and potential future applications of meta-omics in microbiome research. One of the main obstacles is the lack of accessible and user-friendly bioinformatic tools. Currently, these tools are often complex and require a strong background in bioinformatics, limiting their use to researchers with specialized expertise in this area. As a result, the broader scientific community and clinical researchers may face difficulties in utilizing meta-omics data effectively. To address this, it is crucial to develop and expand bioinformatic tools that are more intuitive and accessible to a wider range of researchers. Additionally, reducing the cost of analysis per sample would make these technologies more feasible for widespread use. Together, these improvements could promote the extension of meta-omics applications into diverse fields beyond microbiome research, enabling more widespread and effective use of this data in areas such as personalized medicine, diagnostics, and treatment strategies (Filardo et al., 2024). The Figure 2 showed the Strategies to modify gut microbiota for disease treatment include probiotic-rich dairy, fruits, and beverages, along with powders and microencapsulated forms to enhance digestive and mental health.

## 7 Conclusion and future perspective

The gut microbiota (GM) is deeply interconnected with human health and disease, offering significant opportunities for diagnosing, treating, and preventing various conditions. It is evident that the gut microbiota profoundly influences overall physiology and pathophysiology, as these microorganisms interact with nearly every organ in the body. Probiotics have proven effective in treating infections, gastrointestinal disorders, and inflammatory diseases, and they may also assist in managing obesity and diabetes. Advancements in gut microbiota modeling and analysis will continue to enhance our understanding of its effects on health and disease, facilitating improvements in current and novel strategies for treating and preventing illnesses related to gut microbiota imbalances.

By identifying and comprehending the various functions of the gut microbiome in growth, development, and disease, it is possible to optimize many aspects of health, from infant nutrition to the development of new treatments for conditions like obesity and cancer. Future innovations may include the design of artificial prebiotics and the development of specific, personalized probiotics. Managing dysbiosis often involves microbiota-directed therapies, such as probiotics, prebiotics, smart microbiota modulation, and fecal microbiota transplantation, as alterations in the microbiome are linked to chronic diseases. Further research is required to identify metabolite-producing gut bacteria for use in pharmacology. Additionally, defining the role of microbial communities in gastrointestinal disease pathogenesis is essential to develop effective interventions, including school-based strategies.

Advancing research into microbiome profiling, combined with genomics and metabolomics, will help identify biomarkers for disease prediction and personalized treatment plans. Refining existing therapies like probiotics, prebiotics, and fecal microbiota transplantation (FMT) remains a priority, with the goal of optimizing their efficacy, safety, and clinical application. Emerging therapies, including microbiome-engineered bacteria, bacteriophage therapy, and synthetic biology, open new possibilities for more precise and controlled interventions to restore gut microbial balance. As interest in microbiome manipulation grows, further research on the effects of prebiotics, probiotics, and postbiotics is crucial for improving health outcomes. These therapies hold promise as nonspecific immunomodulators, offering new approaches to regulating gut microbiota composition and potentially predicting patient responses to treatment. While probiotics show therapeutic benefits for gastrointestinal disorders, more evidence is needed to fully define their precise effects. As microbiome research progresses, a deeper understanding of its complex interactions with the immune system and other physiological processes will pave the way for more targeted, personalized microbiome-based treatments, which may eventually complement or replace conventional therapies for gastrointestinal diseases.

## Abbreviations

GI, gastrointestinal; IBD, inflammatory bowel disease; FMT, fecal microbiota transplantation; GBA, gut-brain axis; IECs, intestinal epithelial cells; CRC, colorectal cancer; NAFLDs, nonalcoholic fatty liver diseases; IBDs, inflammatory bowel disorders; CVDs, cardiovascular diseases; GERD, gastroesophageal reflux disease; TJ, tight junctions; PDAC, pancreatic ductal adenocarcinoma; CD, Crohn’s disease; UC, ulcerative colitis; SCFAs, short-chain fatty acids; OS, oxidative stress; MDD, major depressive disorder; CDI, *Clostridium difficile* infection; CNS, central nervous system.
